# Long-term outcomes of edaravone in amyotrophic lateral sclerosis in South Korea: 72-week observational study

**DOI:** 10.1186/s12883-022-02788-x

**Published:** 2022-07-14

**Authors:** Jin-Mo Park, Donghwi Park, Hyung-Jun Kim, Jin-Sung Park

**Affiliations:** 1 Department of Neurology, Dongguk University College of Medicine, Dongguk University Gyeongju Hospital, Gyeongju, Republic of Korea; 2 Department of Physical Medicine and Rehabilitation, Ulsan University hospital, University of Ulsan College of Medicine, Ulsan, Republic of Korea; 3 Dementia Research Group, Korea Brain Research Institute (KBRI), Daegu, 41068 Republic of Korea; 4 Department of Neurology, School of Medicine, Kyungpook National University, Kyungpook National University Chilgok Hospital, 807 Hoguk-ro, Buk-gu, Daegu, 41404 Republic of Korea

**Keywords:** Edaravone, Amyotrophic lateral sclerosis, Adverse effect, Safety

## Abstract

**Background:**

Amyotrophic lateral sclerosis (ALS) is a lethal neurodegenerative disease characterized by the gradual loss of upper and lower motor neurons that leads to progressive muscle atrophy and weakness. Edaravone, a free-radical scavenger, was approved as an ALS treatment in 2015 in South Korea.

**Methods:**

This study investigated the long-term effects and safety of edaravone by reviewing the medical records of 16 Korean patients with ALS who received extended edaravone between 2015 and 2021 in a single tertiary ALS center.

**Results:**

Among sixteen patients, eleven patients underwent extended edaravone therapy for more than 18 cycles (72 weeks). The mean monthly changes in the revised ALS Functional Rating Scale (ALSFRS-R) were − 0.96 ± 0.83 (0–24 weeks), − 0.70 ± 0.76 (24–48 weeks), − 1.18 ± 1.67 (48–72 weeks), and − 0.81 ± 0.60 (0–72 weeks). The mean decline in forced vital capacity (FVC) was 17.4 ± 24.1. The changes were significant in both ALSFRS-R (*p* < 0.001) and FVC (*p* = 0.048); however, the mean change in compound muscle action potential of phrenic nerves was not. Patients experienced only minor adverse events, which were well tolerated.

**Conclusions:**

This study verifies previous reported outcomes of edaravone in 16 Korean ALS patients, indicating a modest effect with a favorable safety profile.

## Background

Amyotrophic lateral sclerosis (ALS) is a lethal neurodegenerative disease characterized by the gradual loss of upper and lower motor neurons, leading to progressive muscle atrophy and weakness of the upper and lower extremities, bulbar palsy, and, eventually, death within 3–5 years due to respiratory failure [1]. Currently, riluzole and edaravone are the only United States Food and Drug Administration (FDA)–approved treatments. Edaravone is a scavenger of peroxyl radicals and perioxynitrites that promotes cell survival against oxidative stress and potentially inhibits apoptosis [2]. It was approved based on a clinical trial involving Japanese patients that demonstrated some benefit in delaying ALS progression and used inclusion criteria based on a post hoc subgroup analysis [3]. In a phase 3, randomized, controlled study of a select group of Japanese ALS patients followed over 6 months, investigators were able to show a significant slowing in the rate of decline in the Revised ALS Functional Rating Scale (ALSFRS-R) scores with edaravone treatment. A recent long-term, open-label, follow-up study showed continued benefit of edaravone up to 48 weeks [4]. However, an Italian study that enrolled 331 ALS patients revealed that edaravone treatment did not show significant differences in disease progression or respiratory function [5]. In addition, concerns have been raised about the generalizability of research results, especially given the selective nature of the inclusion criteria of a relatively short disease duration, preserved respiratory function, and motor function. This study aimed to evaluate edaravone treatment for ALS patients treated for longer period at a single medical center in South Korea.

## Methods

### Patients

This is a retrospective study of patients with ALS who received edaravone infusions in Kyungpook National University Chilgok Hospital from December 2015 to May 2021. All patients were diagnosed as “definite,” “probable,” or “probable laboratory-supported” ALS according to the revised El Escorial criteria [6]. Edaravone was administered to patients with ALS regardless of disease duration, ALSFRS-R item score, or pulmonary function as measured by forced vital capacity (FVC), according to the standard protocol [3]. Data such as age, sex, onset type (limb-onset or bulbar-onset), time of onset of symptoms, BMI, ALSFRS-R score, FVC, riluzole use, the amplitude of the CMAP of the phrenic nerve conduction study, time from symptom onset to ALS diagnosis, time from ALS diagnosis to first infusion, laboratory results (creatinine and albumin), and adverse effects were collected. Medical records were reviewed after informed consent from the patients who participated in edaravone treatment. The study was approved by the Institutional Review Board of Kyungpook National University Chilgok Hospital (KNUCH 2019–03-015).

### Statistical analysis

The data were analyzed using the Statistical Package for the Social Sciences Version 18.0 (SPSS, IBM SPSS Statistics, IBM Corporation, Armonk, NY, USA). Normal distribution of data was assessed using the Kolmogorov–Smirnov test. Data are expressed as mean (standard deviation) and number (proportion). Normally distributed continuous demographic data (baseline and after edaravone treatments) were compared using the paired t-test. Quantitative data were compared between the two groups using Student’s t-test. The Pearson correlation coefficient (*r*) was used to evaluate the correlation between the CMAP amplitude of the phrenic nerve and other clinical data, including BMI, ALSFRS-R, FVC, creatinine, and albumin. Significance level was set at 0.05 *(p* < 0.05).

## Results

**Fig. 1:**
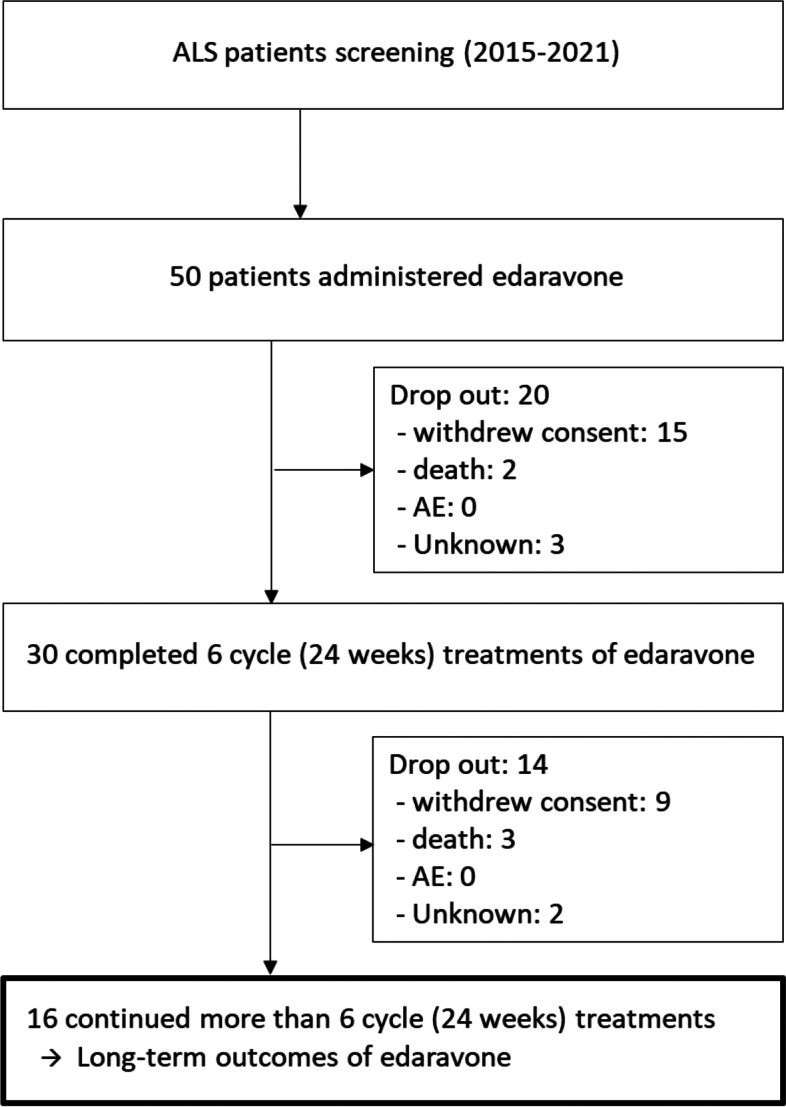
Diagram of the amyotrophic lateral sclerosis patients with edaravone treatment

**Fig. 2:**
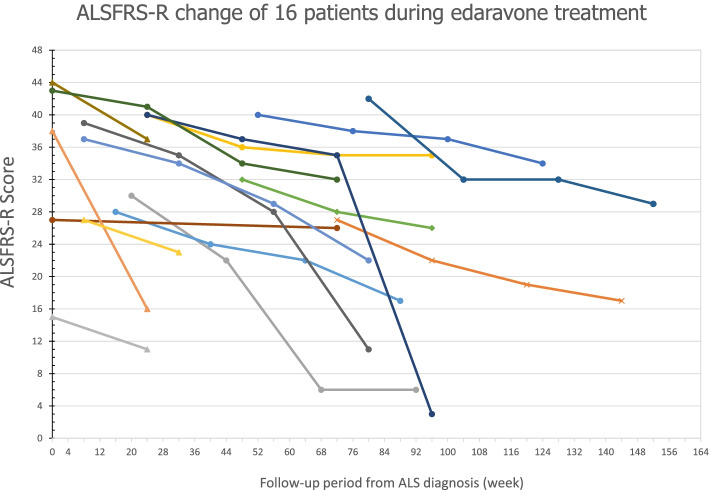
Serial changes in total amyotrophic lateral sclerosis Functional Rating Scale (ALSFRS)-R score during edaravone treatment in 16 patients. Based on the time point from ALS diagnosis, the first mark indicates ALSFRS-R score at the baseline of edaravone treatment; the second mark indicates the score at 24 weeks; the third mark indicates the score at 48 weeks; and the fourth mark indicates the score at 72 weeks. The circle indicates an evaluation that was conducted for 72 weeks, the diamond indicates 48 weeks, the triangle indicates 24 weeks, and the X mark indicates the patient died

Fifty ALS patients participated in edaravone treatment at our tertiary medical center specialized in ALS (Fig. 1), of which 30 completed a 6-cycle administration of edaravone, and 16 agreed to continue receiving an extended treatment (10 men, 6 women; 9 limb-onset, 7 bulbar-onset; mean age, 58.3 ± 10.3 years). Among these 34 drop-out patients, 24 patients dropped as they felt the therapy was ineffective, 5 patients died and 5 patients were unknown due to loss of follow up (Fig. 1). The mean duration of those 20 patients with incomplete treatment was 2.85 cycles (11.4 weeks). The mean time from symptom onset to ALS diagnosis in 16 patients receiving an extended treatment was 14.5 ± 14.03 months, whereas the mean time from ALS diagnosis to first infusion was 8.13 ± 11.13 months. The baseline body mass index (BMI) was 22.80 ± 3.79 kg/m2. Eleven patients who underwent extended edaravone therapy for more than 18 cycles were assessed at 72 weeks of the therapy. The clinical course of the patients progressed at various degrees (Fig. 2).

**Fig. 3:**
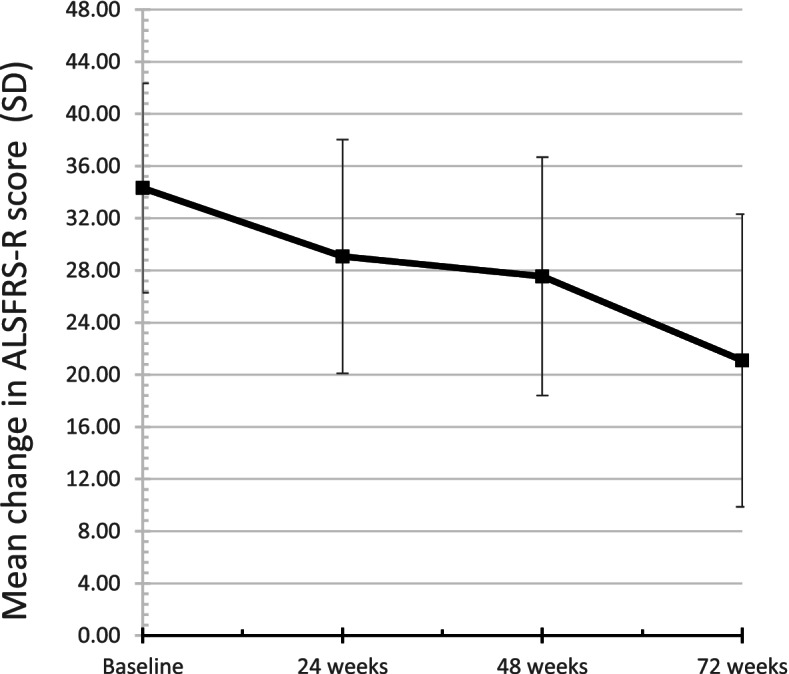
The mean amyotrophic lateral sclerosis Functional Rating Scale ALSFRS-R scores at each time point during edaravone treatment. The baseline score is the mean (standard deviation) ALSFRS-R for 16 patients, the score at week 24 is the score for 15 patients, the score at week 48 is the score for 11 patients, and the score at week 72 is the score for 11 patients

**Fig. 4:**
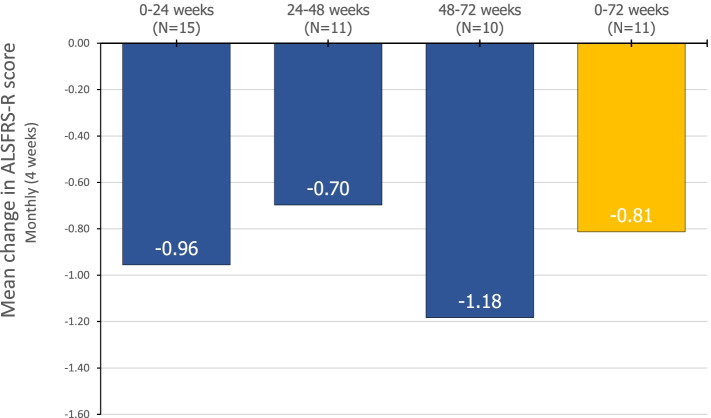
Changes in amyotrophic lateral sclerosis Functional Rating Scale-R scores at each interval

Clinical parameters were assessed at baseline and at 24, 48, and 72 weeks. The mean ALSFRS-R score (Fig. 3) was 34.3 ± 8.3 at baseline (*n* = 16), 29.1 ± 9.0 at 24 weeks (*n* = 15), 27.5 ± 9.1 at 48 weeks (*n* = 11), and 21.1 ± 11.2 at 72 weeks (*n* = 11). The mean FVC (percentage of predicted) was 90.5 ± 20.4 at baseline (*n* = 11) and 72.3 ± 22.9 at follow-up (*n* = 13). The mean monthly change in ALSFRS-R (Fig. 4) was − 0.96 ± 0.83 (0–24 weeks), − 0.70 ± 0.76 (24–48 weeks), − 1.18 ± 1.67 (48–72 weeks), and − 0.81 ± 0.60 (0–72 weeks). The decrease in ALSFRS-R (*p* < 0.001) and FVC (*p* = 0.048) were statistically significant. The amplitude of compound muscle action potential (CMAP) in phrenic nerve conduction study (NCS) was 464.4 ± 293.5 μV (right) and 649.8 ± 463.0 μV (left) at baseline (*n* = 11) and 381.1 ± 346.1 μV (right) and 464.8 ± 447.7 μV (left) at follow-up. The change in the CMAP of the phrenic nerves was not significant.

Next, the correlations among the clinical variables were explored. Baseline age and BMI were assessed. FVC, ALSFRS-R, CMAP in phrenic nerve NCS, creatinine, albumin at baseline, 24, 48 and 72 weeks and their changes between baseline and follow-up (24, 48, or 72 weeks) were analyzed. The progression rate was evaluated with the change of ALSFRS-R score from symptom onset to diagnosis. The progression rate from onset to diagnosis was correlated only with the change in ALSFRS-R during the first interval (baseline to 24 weeks; *r* = − 0.524, *p* = 0.045). Phrenic nerve CMAP was not correlated with FVC or any other clinical factor. Initial albumin level was correlated with BMI (*r* = 0.621, *p* = 0.031) and with ALSFRS-R at 24 weeks (*r* = 0.587, *p* = 0.045). Creatinine at 24 weeks was correlated with ALSFRS-R at baseline (*r* = 0.619, *p* = 0.018), 24 weeks (*r* = 0.613, *p* = 0.020), and 72 weeks (*r* = 0.784, *p* = 0.007). Furthermore, creatinine at 72 weeks was correlated with ALSFRS-R at 72 weeks (*r* = 0.738, *p* = 0.015) and interval change in ALSFRS-R from baseline to 72 weeks (*r* = 0.768, *p* = 0.006). Initial creatinine level was correlated with initial albumin level only.

No significant differences were observed between 9 limb-onset and 7 bulbar-onset patients in clinical variables, including ALSFRS-R, FVC, phrenic NCS, and laboratory results except in albumin level at 48 weeks (*p* = 0.018).

None of the patients treated with extended edaravone therapy experienced any significant adverse events that led to the discontinuation of the treatment. Constipation (*n* = 4), insomnia (*n* = 2), headache (*n* = 1), and transient leukopenia (n = 1) were common adverse events which were well tolerated and remedied with appropriate medications to treat symptoms. The changes in serum creatinine (*p* = 0.104) and albumin (*p* = 0.623) levels showed no significant changes during the treatment. Six patients needed tracheostomy (2 limb-onset and 4 bulbar-onset) and nine needed percutaneous endoscopic gastrostomy tube insertion (3 limb-onset and 6 bulbar-onset). Only one patient in our cohort died after 36 weeks of treatment. He had limb-onset ALS for 27 months from symptom onset to edaravone treatment. Although the patient experienced recurrent aspiration pneumonia, he refused to undergo a gastrostomy and eventually died due to uncontrolled aspiration pneumonia.

## Discussion

ALS is a rare but progressive neurodegenerative disease with an incompletely defined pathophysiology. Currently, the number of disease-modifying therapies that are known to slow the progression of the disease are limited. More than two decades have passed since the glutamate inhibitor riluzole was approved for the treatment of ALS after it was demonstrated to extend survival to an average of 2 to 3 months longer when compared with placebo [7]. In 2017, the potent antioxidant edaravone was approved by the U.S. FDA for use in ALS [3]. It was subsequently approved in South Korea and was shown to have moderate effects in a study in Korean ALS patients [1].

Recent studies of edaravone have been conducted on patients with relatively early and mild ALS, demonstrating the efficacy of edaravone therapy in these patients [3, 4]. However, other results cast doubt on the efficacy of edaravone in ALS patients under similar conditions [5]. Despite the disparities reported on the efficacy of edaravone treatment, all the studies commonly acknowledge the safety of edaravone therapy. Still, few studies have reported efficacy and safety of extended edaravone therapy. A recent study showed the safety of long-term treatment in ALS patients for 48 weeks [4], reporting a decrease of 8.0 points and an average decrease of approximately 0.67 points per 4 weeks in the edaravone-treated group. In our study, the average decrease was 0.81 points every for 4 weeks for 72 weeks. This result may be attributed to the differences in disease duration and severity of ALS at baseline. Furthermore, the Korean national insurance policy currently does not cover edaravone treatment, which indicates that most of the patients enrolled would have relatively more advanced disease at initial enrollment. In addition, Okada et al. reported that their projected placebo group showed a decrease of 13.0 points in 48 weeks (1.1 points per 4 weeks) [8]. Comparing our results with this placebo group, edaravone treatment might result in a modest improvement in more advanced ALS, as observed in our previous study. In the present study, the ALSFRS-R score decreased by 0.96 point from baseline to week 24, by 0.70 point from week 24 to week 48, and by 1.18 points from week 48 to week 72. This reflects the heterogeneity in the non-linear progression of ALS [4]. However, caution should be observed in the interpretation of these results due to the small sample size and heterogeneous progression and deterioration of the disease in the study sample.

Most of the recent clinical trials used pulmonary function test (PFT) as an evaluation of respiratory symptoms. However, in clinical practice, with the progression of ALS, getting the appropriate PFT data would be challenging with the aggravation of bulbar muscle weakness. To demonstrate this actual clinical issue, we also encountered five patients who were unable to undergo a PFT due to progression of bulbar weakness. To overcome this limitation, we additionally evaluated respiratory function using the phrenic NCS, which indicated stable pulmonary function among the ALS patients in this longitudinal study. However, we found no statistically significant relationship between FVC and phrenic NCS. However cautious interpretation is needed as our study included a small number of ALS patients and more studies are needed to elucidate the relationship between FVC and phrenic nerves. More studies are needed to establish the usefulness of phrenic nerve study in ALS.

The creatinine and albumin are known surrogate laboratory markers that reflect ALS progression [9, 10]. Previous studies have shown that low albumin and creatinine levels strongly correlate with clinical deterioration as indicated by the ALSFRS-R score and PFT. A recent study showed that, although albumin levels were correlated with the clinical status, it was not correlated with the BMI, indicating that albumin is independent of nutritional status but is a marker of inflammation [9]. However, the creatinine level correlated with the BMI, indicating malnutrition in ALS. In our study, BMI was correlated with albumin, not creatinine. Furthermore, whereas creatinine was associated with ALSFRS-R throughout the clinical course, albumin was only associated with ALSFRS-R at the beginning of treatment.

This study has a few limitations that need to be addressed. First, this was a retrospective study, and patients who received edaravone treatment were enrolled consecutively without a control group. Second, since ALS is a heterogeneous disease that features a varied clinical course, some patients will possibly have a comparatively slower disease progression. Third, this study enrolled patients with advanced-stage ALS; as this was an observational study, the findings of this would not be applicable to early-stage ALS patients. Furthermore, due to the rapid progression of the disease compared with other neurodegenerative diseases, the collection of sufficient clinical data and sampling was limited.

## Conclusions

This is the first Korean study that describes the long-term clinical course of sixteen ALS patients treated with edaravone, who were followed up for 72 weeks. Our results show that long-term treatment with edaravone was well tolerated with no significant adverse events that led to the discontinuation of the treatment. Moreover, modest improvement was observed in advanced ALS patients. Thus, a 72-week treatment with edaravone can be considered safe with modest improvement. Nevertheless, further well-designed studies are warranted to clarify the effect of edaravone and its possible adverse events after extended therapy.

## Data Availability

The datasets generated and/or analysed during the current study are not publicly available due to requirement for patient confidentiality, but are available from the corresponding author on reasonable request.

## Abbreviations

ALS: Amyotrophic lateral sclerosis
FDA: Food and Drug Administration
ALSFRS-R: Revised ALS Functional Rating Scale
FVC: Forced vital capacity
BMI: Body mass index
CMAP: Compound muscle action potential
NCS: Nerve conduction study
PFT: Pulmonary function test
